# Atherosclerotic cardiovascular diseases in inflammatory bowel diseases: to the *heart* of the issue

**DOI:** 10.3389/fcvm.2023.1143293

**Published:** 2023-05-16

**Authors:** Roberto Gabbiadini, Arianna Dal Buono, Elisabetta Mastrorocco, Virginia Solitano, Alessandro Repici, Antonino Spinelli, Gianluigi Condorelli, Alessandro Armuzzi

**Affiliations:** ^1^ IBD Center, Department of Gastroenterology, IRCCS Humanitas Research Hospital, Milan, Italy; ^2^ Department of Biomedical Sciences, Humanitas University, Milan, Italy; ^3^ Division of Colon and Rectal Surgery, IRCCS Humanitas Research Hospital, Milan, Italy; ^4^ Department of Cardiovascular Medicine, IRCCS Humanitas Research Hospital, Milan, Italy

**Keywords:** inflammatory bowel disease, atherosclerotic cardiovascular disease, ischemic heart disease, ischemic stroke, IBD therapy

## Abstract

Atherosclerotic cardiovascular disease and stroke are the leading causes of morbidity and mortality worldwide. Along to the traditional risk factors for these diseases, chronic inflammation is known to be an important player in accelerating the process of atherosclerosis, which can result in an increased incidence of arterial thromboembolic events. As in other chronic inflammatory diseases, in the past few years, several studies suggested that subjects affected by inflammatory bowel diseases (IBD) may also be at an incremented risk of atherosclerotic disease, especially during the periods of disease's flare. Therefore, IBD treatment may assume an important role for achieving both disease remission and the control of the atherosclerotic risk. In this article we aimed to perform a comprehensive review on evidence on the increased risk of arterial thromboembolic events in patients affected by IBD and discuss the potential role of IBD therapy in reducing this risk.

## 1. Introduction

Inflammatory bowel diseases (IBD), specifically Crohn's disease (CD) and ulcerative colitis (UC), are a group of life-long disorders characterized by chronic relapsing inflammation of the gastrointestinal tract (1, 2). The pathogenesis of IBD is not yet fully understood. CD and UC are immune-mediated diseases that originate from an abnormal immune response to the gut microbiota in genetically susceptible hosts (3). Therefore, the inflammation of the intestinal mucosa leads to the development of symptoms such as diarrhea, abdominal pain, bloody stools, and weight loss (4). In addition to gastrointestinal symptoms, CD and UC can also be associated with various immune-mediated extraintestinal manifestations which can affect the joints, skin, or eyes and whose mechanisms can be ascribed to an extension of the intestinal immune response or an independent inflammatory event perpetuated by IBD (5, 6). Furthermore, similar to other immune-mediated diseases, IBD can also be associated with other extraintestinal comorbidities which are a direct or indirect result of bowel inflammation and that are linked to a reduced quality of life and outcomes due to hospitalization, surgery complications, and mortality (5, 7). The perception of comorbidities in IBD is recently emerging and, in the late years, there has been plentiful interest on this topic (7). Among extraintestinal comorbidities, atherosclerotic cardiovascular disease (ASCVD) is one of the most relevant (7). Indeed, ASCVD remains the leading cause of morbidity and mortality worldwide (8). IBD has so far been overlooked as a risk factor for ASCVD, since, similarly to other chronic inflammatory diseases (i.e., rheumatoid arthritis), chronic inflammation is a main driver for the development of accelerated atherosclerosis and some studies suggest that IBD may display an incremented risk for arterial thrombotic events (9–11).

The aim of this review is to highlight the current evidence on the increased risk of ASCVD in patients affected by IBD, and discuss the shared physiopathology, present and future therapeutical implications.

## 2. Methods

We carried out a literature search in the PubMed database until November 2022 to identify relevant studies investigating the shared mechanisms and association between ASCVD and IBD. The key words used were: “inflammatory bowel disease” OR “Crohn's disease” OR “ulcerative colitis” AND “cardiovascular disease” OR “atherosclerosis” OR “ischemic heart disease” OR “ischemic stroke”. In addition, references of original articles and relevant reviews were screened to find additional publications. No publication date restrictions were applied. We excluded case reports, case series and any irrelevant abstract.

## 3. Pathological mechanisms shared by IBD and atherosclerotic cardiovascular diseases

**Figure 1:**
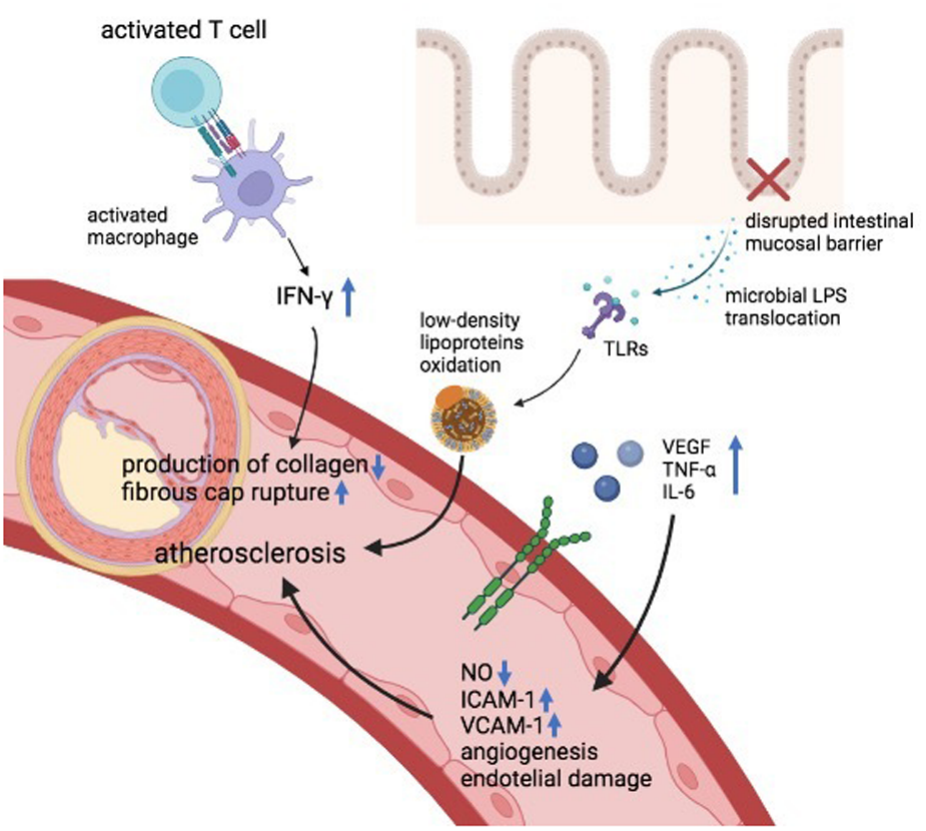
Pathological mechanisms shared by IBD and atherosclerotic cardiovascular diseases. IFN-γ, interferon gamma, TLRs, toll-like receptors, VEGF, vascular-endothelial growth factor, TNF-α, tumor necrosis factor, IL, interleukin, NO, nitric oxide, ICAM, intercellular adhesion molecule, VCAM, vascular cell adhesion protein, LPS, lipopolysaccharides.

Chronic inflammation and inflammatory cytokines displayed by chronic immune-mediated diseases have been pointed as important players in the pathogenesis of atherosclerosis and rapid coronary arterial disease evolution (12). For its being chronic inflammatory diseases, also IBD are associated with an upregulation of several cytokines (13). It has been shown that IBD patients with active disease, compared to subjects with quiescent disease and healthy controls, exhibit higher levels of vascular endothelial growth factor (VEGF) which is a potent angiogenic and vascular permeability-enhancing cytokine (14–16). Also, other inflammatory molecules that have an important role in the pathogenesis of IBD, such as tumor necrosis alpha (TNF-α), can generate endothelial alterations and can damage vascular functionality by reducing the nitric oxide (NO) availability (16, 17). Furthermore, TNF-α promotes the interconnection between the endothelium and circulating leukocytes by the upregulation of adhesion molecules such as vascular cell adhesion molecule 1 (VCAM-1) and intercellular adhesion molecule 1 (ICAM-1) (10). High levels of VCAM-1 and ICAM-1 have been described as an independent risk of major adverse cardiac events (18). The expression of ICAM-1 and VCAM-1 is associated with intestinal disease activity, demonstrated by the reduction of their serum concentration after treatment (10, 19). In addition, interleukin-6 (IL-6), whose levels are increased in IBD, is linked to endothelial dysfunction, early atherosclerosis, and coronary heart disease (20–23). Moreover, both innate and adaptive cells are involved in the linkage between IBD and atherogenesis (16). It has been acknowledged that in IBD, the disruption of the intestinal mucosal barrier promotes the translocation of microbial lipopolysaccharides (LPS) that can stimulate the production of proinflammatory molecules and the oxidation of low-density lipoproteins through Toll-like receptors (TLRs) signaling, leading to endothelial injury and atherosclerosis (24). TLRs are fundamental in both IBD and atherosclerosis pathogenesis and have been observed in atherosclerotic plaques (16, 24). LPS as well are present in atherosclerotic arteries but not in normal arteries. In atherosclerotic plaques, the pro-inflammatory status induced by LPS can also lead to plaque instability (25). Beyond the innate immune system, also the adaptative immune system plays an important role in the network between IBD and atherogenesis. IBD patients are characterized by activated T cells (26) which are crucial also in the pathogenesis of atherosclerosis (16, 27). Indeed, activated T cells has been observed in atherosclerotic lesions (16), similarly as in Crohn's disease. The T cells infiltrating atherosclerotic plaques exhibit a Th1 profile which activates macrophages and expands pro-inflammatory cytokines such as IFN-γ. IFN-γ reduces the production of collagen, making the atherosclerotic fibrous cap more susceptible to rupture (27). Also genetics factors are implicated in the pathogenesis of IBD and atherosclerosis and some gene variants may be shared by both diseases (16). Indeed, it has been observed that some nucleotide-binding oligomerization domain-containing protein 2 (NOD2)/CARD15 polymorphisms (2 missense mutations: ARG702TRP, GLY908ARG; 1 frameshift mutation: 1,007 fs) implicated in the regulation of CD were associated also with the development of coronary atherosclerosis (28–30). Conversely, a polymorphism of IL-6 receptor (rs2228145) seems to possess a protective role in both coronary heart disease and IBD (31). In the future, more studies are needed to assess the role of these or other genetic variants in the predisposition to atherosclerosis in individuals with IBD. Finally, the alterations in the gut microbiota of IBD patients and the microbiota-derived processes may be associated to atherosclerosis (32). Interestingly, an increased microbiota composition of Enterobacteriaceae species (mainly Escherichia coli) have been described in both IBD and cardiovascular disease even if the connection between these findings have not been cleared (32–34). Furthermore, butyrate-producing bacteria (i.e., Faecalibacterium prausnitzii) can be decreased in the microbiota of either IBD and CVD individuals and it is known that butyrate possess an atheroprotective role and can hamper the intestinal epithelial cell apoptosis, therefore reducing LPS translocation (32, 35–37). Figure 1 summarizes the shared pathological mechanisms between IBD and ASCVD.

## 4. Risk of ischemic heart disease in IBD

Several studies assessed the interconnection between IBD and the risk of ischemic heart disease (IHD) with heterogeneous results. Although some retrospective studies did not find a significant association between IBD and IHD (38–41), many other studies and meta-analyses demonstrated a positive correlation between the two diseases (11, 42–44). A landmark population-based matched-cohort study conducted by Bernstein et al. (8,072 IBD patients and 80,489 non-IBD controls) found an increased incidence rate ratio of IHD in the IBD cohort (IRR: 1.26; 95% CI: 1.11–1.44) (45).

Subsequently, also a large nationwide Danish population-based cohort study (4,570,820 people, 28,833 with IBD) showed that IBD patients have an increased risk of IHD in the first year after IBD diagnosis (IRR = 2.13, 95% CI: 1.91–2.38) compared to IBD-free individuals. Furthermore, this study showed that in the long term, during 1–13 years of follow-up, the risk was 1.22 (95% CI: 1.14–1.30) (46). Similarly, a more recent large population-based study from the United States (29,090,220 people, 290,430 with IBD) found that IBD confer a higher risk of myocardial infarction (adjusted odds ratio: 1.25; 95% CI: 1.24–1.27) (47). Comparable results were noted also in a longitudinal cohort study with matched controls conducted by Yarur et al. (48). In this study, 356 IBD patients and 712 controls were followed for a median of 53 and 51 months, respectively. An increased incidence of coronary artery disease was observed in subjects with IBD (adjusted hazard ratio: 4.08; 95% CI: 2.49–6.70) (48). Interestingly, it was also found that IBD patients had lower rates of traditional coronary artery disease risk factors (hypertension, diabetes, dyslipidemia, and obesity) (48). These findings were corroborated by other studies. The study of Aniwan et al., showed that IBD was independently associated with an increased risk of acute myocardial infarction (AMI) (adjusted hazard ratio: 2.82; 95% CI: 1.98–4.04), despite a lower prevalence of conventional risk factors for AMI (49). Similar results were observed also by Aarestrup et al. (50). In their population-based study of >100.000 individuals (1,203 with IBD), the Authors observed that, even though subjects with IBD were diagnosed more frequently with cardiovascular diseases (13.2% vs. 10.9%; *p* = 0.009), conventional cardiovascular risk factors were not increased. Furthermore, among subjects with IBD, those who developed cardiovascular diseases displayed higher levels of C-reactive protein (CRP) (50). In addition, another study found that IBD patients diagnosed with coronary artery disease (CAD) were younger, less active smokers and had lower body mass index compared to non-IBD patients diagnosed with CAD, despite similar prevalence of hypertension, hyperlipidemia and diabetes was exhibited in both groups (51). This trend was observed as well by Haapamäki et al., which showed an increased frequency of coronary heart disease (CHD) in subjects with IBD compared to controls (2.2% vs. 1.4%; *p* = 0.004) despite a younger age of the IBD group (52). Again, in another nationwide study, Choi et al. observed that the risk of myocardial infarction was higher in subjects with CD than in the general population (hazard ratio: 1.80; 95% CI: 1.47–2.21) and that this tendency was stronger in female patients and those ones aged under 40 years (53). Similarly, Nasir et al. demonstrated an increased odds of ASCVD in IBD compared to non-IBD (odds ratio: 1.58; 95% CI: 1.17–2.13) with a significant interaction by age whereby the younger people displayed a stronger association with ASCVD (adjusted OR among 18–44 year-olds 3.35; 95% CI: 1.75–6.40) (54). These evidences reinforce the hypothesis that conventional cardiovascular risk factors might not be enough to estimate the risk of CAD in subjects with IBD and that intestinal chronic inflammation likely play a crucial role in the pathogenesis of atherosclerosis (50, 51). Indeed, several studies demonstrated a positive relation between CRP, an important quantifiable marker of systemic inflammation, and the risk of IHD or acute arterial events (50, 55, 56).

Likewise, a large nationwide French cohort study (*n* = 210,162 IBD patients) conducted by Kirchgesner et al. observed that disease activity was associated with an independent increased risk of IHD in both CD (hazard ratio: 1.59; 95% CI: 1.23–2.04) and UC (hazard ratio: 1.94; 95% CI: 1.54–2.44) (57). Other several studies showed that acute coronary disease was correlated to IBD disease activity (58–61). Therefore, a recent international consensus stated that IBD patients, especially young subjects with active disease, harbor an increased risk of arterial thrombotic events, recommended to strive for intestinal disease remission in order to reduce its atherosclerotic risk and suggested to perform an active screening and control of traditional cardiovascular disease risks in the setting of IBD (11). Interestingly, a meta-analysis conducted by Sun et al. (42) observed a higher relative risk of CHD in women with IBD compared to the increment of the relative risk in male with IBD (RR: 1.27; 95% CI: 1.12–1.45 vs. RR: 1.13; 95% CI: 1.09–1.17, respectively) and this may be partially explained by the variance in the gender distribution of traditional IHD risk factors and by the fact that inflammation may play a higher role in women (62, 63). Furthermore, the same meta-analysis also observed that subjects with IBD were not associated with an increased cardiovascular mortality. Possible explanations for this result is that cardiovascular mortality may not be a good surrogate for IHD incidence and that the advancement of medical therapy and health care may reduce the mortality from cardiovascular diseases (42). Further prospective studies are needed to evaluate if the increased incidence of IHD in IBD will also result in an increased cardiovascular mortality. Table 1 summarizes the studies which evaluated the risk of IHD in IBD.

**Table 1:** Summary of the studies exploring the risk of ischemic heart disease in IBD.

Authors	Year of publication	Study design	Results
Sun et al. (42)	2018	Meta-analysis	11 studies which reported the risk of cardio-vascular disease incidence were analyzed. The pooled relative risks of coronary heart disease and MI among IBD patients compared with those without IBD was 1.17 (95% CI: 1.07–1.27)
Li et al. (43)	2021	Meta-analysis	14 studies (including 710,250 IBD patients and 5,671,535 healthy controls) were included. IBD was associated with higher risk of IHD (OR/RR: 1.26, 95% CI: 1.20–1.32). Both UC and CD were associated with higher risk of IHD (UC: OR/RR: 1.19, 95% CI: 1.13–1.26; CD: OR/RR: 1.33, 95% CI: 1.17–1.51).
Feng et al. (44)	2017	Meta-analysis	10 cohort studies were included. Subjects with IBD were associated with an increased risk of IHD (RR: 1.244; 95% CI, 1.142–1.355).
Osterman et al. (38)	2011	Retrospective cohort study	15,498 subjects with UC were matched to 144,605 general practice patients and 9,829 subjects with CD were matched to 92,987 patients from general practice. Neither patients with UC or CD had a significantly increased risk of acute myocardial infarction (UC: adjusted HR: 1.11, 95% CI: 0.98–1.27; CD: adjusted HR, 1.09, 95% CI: 0.89–1.34).
Barnes et al. (39)	2016	Retrospective cross-sectional study	567,438 hospitalizations for MI among subjects with IBD and 78,121,000 among the general population. In adjusted analyses, IBD patients had lower rates of hospitalizations for MI compared with the general population (odds ratio, 0.51; 95% CI: 0.50–0.52).
Gill et al. (40)	2021	Observational study	Among patients with IBD (*n* = 15,292), incidence of MI did not show a statistically significant difference (HR: 1.05, 95% CI: 0.89–1.23) when compared to the matched cohort (*n* = 30,584)
Setyawan et al. (41)	2022	Retrospective cohort study	To assess the incremental rates of MI among IBD patients, subjects with IBD were matched to subjects without IMD (*n* = 34,687 each cohort). No significant increase in rates was observed for MI: (adjusted IRRs: 0.62; 95% CI: 0.44, 0.88).
Bernstein et al. (45)	2008	Population-based cohort study	An IBD cohort (*n* = 8,060) and a matched non-IBD cohort (*n* = 80,489) were compared for the incidence of IHD. The risk was increased for all IBD (IRR: 1.26; 95% CI: 1.11–1.44) and for both CD and UC.
Rungoe et al. (46)	2013	Population-based cohort study	In a nationwide population-based cohort of 4,570,820 people, subjects with IBD (*n* = 28,833) were compared with IBD-free subjects. IBD patients had an increased risk of IHD in the first year after IBD diagnosis (IRR = 2.13, 95% CI: 1.91–2.38) compared to IBD-free individuals.
Panhwar et al. (47)	2019	Population-based cohort study	Out of 29,090,220 subjects, 131,680 had UC and 158,750 had CD. After adjusting for age, race, gender, and conventional cardiovascular risk factor, IBD conferred greater odds of MI (adjusted OR: 1.25, 95% CI: 1.24–1.27).
Yarur et al. (48)	2011	Longitudinal cohort study	356 subjects with IBD and 712 matched controls were followed for a median of 53 and 51 months. Despite a lower rates of conventional CAD risk factors (hypertension, diabetes, dyslipidemia, and obesity) among IBD patients, they displayed an increased incidence of CAD (adjusted OR: 4.08, 95% CI: 2.49–6.70).
Aniwan et al. (49)	2018	Longitudinal cohort study	736 IBD patients and 1,472 controls were identified. IBD associated independently with increased risk of MI (adjusted HR: 2.82; 95% CI: 1.98–4.04); both CD (aHR vs. controls, 2.89; 95% CI: 1.65–5.13) and UC (aHR vs. controls, 2.70; 95% CI: 1.69–4.35).
Aarestrup et al. (50)	2019	Population-based cohort study	108,789 participants were included (1,293 with IBD). Subjects with IBD were more frequently diagnosed with CVD (13.2% vs. 10.9%; *p* = 0.009); however conventional cardiovascular risk factors were not increased. IBD individuals who developed CVD had a higher level of hs-CRP (median [IQR] = 2.3 [1.2–4.2] mg/L) compared to those who did not developed CVD (median [IQR] = 1.5 [1.0–2.9] mg/L)
Aggarwal et al. (51)	2014	Historical cohort study	131 IBD with CAD and 524 matched non-IBD controls with CAD were included. Subjects with IBD were younger (65.3 ± 10.0 vs. 67.8 ± 11.0 year, *p* = 0.016), were less active smokers (10.7% vs. 18.7%, *p* = 0.03), and had lower body mass index (28.0 ± 5.1 vs. 29.4 ± 6.4, *p* = 0.026) compared with controls.
Haapamäki et al. (52)	2011	Retrospective cohort study	2,831 IBD patients and 5,662 control patients were recruited. CHD occurred more frequently in IBD individuals than in controls (OR: 1.883; 95% CI: 1.297–2.733; *p* = 0.004). Active disease was a risk factor for CHD (*p* = 0.018).
Choi et l. (53)	2019	Nationwide cohort study	10,708 individuals with CD and 26,769 with UC were recruited. 112,431 general population controls were compared with IBD cohort. MI risk was higher in CD than in controls (HR, 1.80; 95% CI: 1.47-2.21). This was more evident in subjects aged <40 years (HR, 2.96; 95% CI: 1.96-4.47). Female patients with UC had an increased risk of MI (HR, 1.33; 95% CI: 1.13-1.56).
Nasir et al. (54)	2022	Retrospective cross-sectional analysis	66,610 surveyed participants were included (951 with IBD and 165 with ASCVD). In multi-variable analyses adjusting for age, sex, ethnicity and traditional risk factors, IBD was associated with an OR for ASCVD of 1.58 (95% CI: 1.17–2.13). This association was stronger in individuals with lower age (OR: 3.35; 95% CI: 1.75–6.40 among 18–44 years old).
Fang et al. (55)	2022	Retrospective cohort study	1,435 patients with IBD were matched with 1,588 individuals without IBD. Subjects with IBD had higher incidences of IHD than matched controls (12.1% vs. 5.5%; *p* < .001). The risk of IHD reached a peak level in patients aged 18–35 years in IBD compared with non-IBD subjects (CD:OR: 6.33, 95% CI: 3.29–12.16; UC: OR: 3.00, 95% CI: 1.18–7.60). CRP was positively related with the risk of IHD (OR, 1.02; 95% CI: 1.01–1.03) in individuals with CD.
Alayo et al. (56)	2022	Retrospective cohort study	5,094 subjects with IBD were matched to 20,376 non-IBD controls. Individuals with IBD had a higher risk of AAEs (aHR, 1.19; 95% CI: 1.08–1.31). hs-CRP (highest quartile; aHR, 1.53; CI, 1.15–2.03) and disease severity (aHR, 5.40; CI, 4.03–7.22) were independent predictors of AAE in IBD.
Kirchgesner et al. (57)	2018	Nationwide cohort study	210,162 individuals with IBD were identified (5,554 with AAEs). The risk of AAEs and IHD were increased in subjects with IBD compared with the general population (SIR, 1.19; 95% CI: 1.16–1.22; SIR, 1.17; 95% CI: 1.13–1.21, respectively). The highest risk was observed in subjects with age <55 years, both in CD and UC. Disease activity was associated with an independent increased risk of IHD in both CD (hazard ratio: 1.59; 95% CI: 1.23–2.04) and UC (hazard ratio: 1.94; 95% CI: 1.54–2.44).
Kristensen et al. (58)	2013	Nationwide cohort study	20,795 patients with IBD were matched to 199,978 controls. Subjects with IBD had an increased risk of MI (rate ratio: 1.17, 95% CI: 1.05–1.31). During flares the rate ratios of MI increased to 1.49 (95% CI: 1.16–1.93). During remission the risk of MI was similar to controls.
Tsai et al. (59)	2014	Nationwide cohort study	11,822 individuals with IBD symptoms were matched to 47,288 controls patients without disorder. The incidence of ACS was 87% higher in the IBD cohort than in the control cohort. The adjusted HRs of ACS for the patients with IBD was 1.72 (95% CI: 1.53–1.94). Subjects with IBD who required 2 or more hospitalization per year (p/y) were nearly 20-fold more likely to have ACS than those who needed 1 hospitalization p/y.
Le Gall et al. (60)	2018	Nested case-control study	30 IBD individuals with AAEs (IS or ACS) and 60 matched controls (IBD without AAEs) were included. In the multivariate analysis, clinical disease activity was significantly associated with the risk of AAEs (OR: 10.4, 95% CI: 2.1–49.9).
Card et al. (61)	2021	Retrospective cohort study	31,175 IBD individuals and 154,412 matched controls were recruited. In Cox regression–adjusted models for potential confounders, no significant excess of vascular events for subjects with IBD. However, an increased risk of MI for acute disease (HR: 1.83, 95% CI: 1.28–2.62) and chronic activity (HR: 1.69, 95% CI: 1.24–2.30) was observed.

AAEs, acute arterial events; ACS, acute coronary syndrome; ASCVD, atherosclerotic cardiovascular disease; CAD, coronary artery disease; CD, Crohn's disease; CI, confidence interval; CHD, coronary heart disease; CRP, C-reactive protein; CVD, cardiovascular disease; HR, hazard ratio; IBD, inflammatory bowel disease; IHD, ischemic heart disease; IMD, immune-mediate disease; IQR, interquartile range; IRRs, incidence rate ratios; IS, ischemic stroke; MI, myocardial infarction; OR, odds ratio; RR, relative risk; SIR, standardized incidence ratio; UC, ulcerative colitis.

## 5. Risk of cerebrovascular ischemic disease in IBD

Stroke is the second leading cause of both disability and death worldwide (64) and arterial occlusion represents its etiology in 87% of cases (65). Similarly to IHD, also a higher risk of stroke has been associated with chronic inflammatory diseases (66, 67), and to increased concentrations of systemic inflammatory factors such as CRP (68). These findings suggest that chronic inflammation may as well play a relevant role in the development of cerebrovascular ischemic accidents. Indeed, IBD patients have evidence of premature cerebrovascular disease (10). An interesting study conducted by Biondi et al. observed that IBD patients had a 6.45-fold higher risk of carotid atherosclerotic plaque compared to healthy controls (95% CI: 1.035–40.216; *p* < 0.046) (69). These findings may have an implication in the daily clinical practice as a large population-based study (52,176,550 patients, 261,890 with IBD) showed that IBD is an independent risk factor for cerebrovascular accidents (OR: 8.07, 95% CI: 7.9–8.2) (70). Another population-based cohort study recruiting 18,392 IBD patients and 73,568 matched controls showed that the risk of ischemic stroke was 1.12-fold higher among the IBD cohort (95% CI: 1.02–1.23) (71). Similarly, Keller et al. observed that CD patients had a higher hazard ratio (HR) for stroke compared to non-IBD patients (HR: 1.91; 95% CI: 1.65–2.22) (72) and this was supported also by the results achieved in the study of Tanislav et al. (HR: 1.50; *p* = 0.013) (73). In accordance with the above-mentioned studies, a recent meta-analysis of cohort and case-control studies confirmed that IBD was associated with an elevated risk of stroke (OR/RR: 1.21; 95% CI: 1.08–1.34). Furthermore, both CD and UC were significantly associated with stroke (CD: OR/RR: 1.25. UC: OR/RR: 1.09) (74). Likewise as IHD, studies showed that especially younger patients display an increased risk of ischemic stroke (75, 76) and that the risk of stroke is significantly increased during IBD flares but not during remission (77). Furthermore, it seems that the risk of incidence of stroke is higher in female IBD patients compared to male ones, as it was observed in the metanalysis by Yuan et al. (RRs of stroke incidence: men 1.23, 95% CI: 1.04–1.45; women 1.46, 95% CI: 1.12–1.91) (78). Gender differences in the risk of stroke may be explained by a major role of systemic inflammation and hormonal discrepancy in women and by the fact that men have a higher background risk which could exceed the independent risk of IBD for stroke (11). Finally, it is important to note that also atrial fibrillation (AF), which incidence is increased in IBD, may partially contribute to the increased risk of stroke in IBD patients (77). However, in the nationwide study of Kristensen et al., the relative impact on stroke risk attributable to AF was significantly lower among individuals with IBD compared with the matched controls (77). Table 2 summarizes the studies which evaluated the risk of cerebrovascular ischemic disease in IBD.

**Table 2:** Summary of the studies exploring the risk of cerebrovascular ischemic disease in IBD.

Authors	Year of publication	Study design	Results
Chen et al. (74)	2021	Meta-analysis	8 cohort studies and 1 case–control study (including 149,908 patients with stroke) were identified. IBD was associated with an elevated risk of stroke (OR/RR = 1.21, 95% CI: 1.08–1.34). Both CD and UC were associated with a higher risk of stroke (CD: OR/RR = 1.25, 95% CI: 1.03–1.52; UC: OR/RR = 1.09, 95% CI: 1.04–1.15).
Xiao et al. (76)	2015	Meta-analysis	8 articles (126,493 IBD patients and 4,748 cases of stroke) were included. IBD had an increased risk of stroke (HR: 1.29; 95% CI: 1.16–1.43). The risk of stroke was higher in younger individuals (HR: 1.48; 95% CI: 1.77–2.85) than in older individuals (HR = 1.35; 95% CI: 1.04–1.77)
Yuan et al. (78)	2015	Meta-analysis	8 cohort studies were included. IBD patients experienced an increased risk of stroke when compared with non-IBD (combined RR: 1.32; 95% CI: 1.20–1.44). The pooled estimate of multivariate RRs was 1.23 among men (95% CI: 1.04–1.45), and 1.46 among women (95% CI: 1.12–1.91).
Ghoneim et al. (70)	2020	Population-based cohort study	52,176,550 subjects were included, of whom 261,890 with IBD. The prevalence of CVA was higher in individuals with IBD compared to non-IBD patients (6.24% vs. 0.48%, *p* < 0.0001). After adjusting for conventional risk factors for CVA, the OR of CVA in subjects with IBD remained higher (OR: 8.07, 95% CI: 7.9–8.2).
Huang et al. (71)	2014	Retrospective cohort study	18,392 patients with IBD and 73,568 matched non-IBD control patients were included. The risk of IS was 1.12-fold (95% CI: 1.02–1.23) higher among IBD individuals than among non-IBD individuals.
Keller et al. (72)	2015	Population-based cohort study	A cohort of 3,309 subjects with CD and a comparison cohort of 13,236 matched non-IBD individuals were included. The HR for stroke among the CD cohort was 1.911 (95% CI: 1.65–2.22) compared to the non-IBD cohort.
Tanislav et al. (73)	2021	Petrospective cohort study	Each cohort (subjects with IBD and non-IBD matched controls) included 11,947 individuals. Stroke and TIA incidences were higher in CD patients than in controls (stroke: HR: 1.50, *p* = 0.013; TIA: HR: 1.93, *p* = 0.004). No relevant differences in were found in UC patients.
Andersohn et al. (75)	2010	Population-based nested case-control study	8,054 patients with and 161,078 patients without CD were recruited. 1,748 cases of IS were identified to whom 17,348 controls were matched. CD was not associated with an increased risk of IS (OR: 1.10, 95% CI: 0.85–1.43). However, an increase in risk was observed in younger patients (<50 years: OR: 2.93; 95% CI: 1.44–5.98) but not in elderly patients (> or =50 years: OR: 0.99; 95% CI: 0.75–1.30).
Kristensen et al. (77)	2014	Nationwide cohort study	24,499 subjects with IBD and 236,275 matched controls were recruited. Increased stroke risk was exclusively found during active IBD (IRRs for flares: 1.57, 95% CI: 1.27–1.93) (IRRs for persistent activity: 1.71, 95% CI: 1.32–2.21) (IRRs for remission: 1.04, 95% CI: 0.93–1.15).

CD, Crohn's disease; CVA, cerebrovascular accidents; IRRs, incidence rate ratios; IS, ischemic stroke; TIA, transient ischemic attack; RR, relative risk; UC, ulcerative colitis.

## 6. IBD therapies and cardiovascular risk

The increased risk of arterial thrombosis in IBD must be taken into account also during the prescription of treatment, because the risk of thrombosis may be affected by these therapies (11, 16) as some IBD drugs display an intrinsic pro or anti-thrombotic effect. One of the most common therapy used in IBD is 5-aminosalicylic acid (5-ASA). This drug shares many pharmacological features of non-steroidal anti-inflammatory drugs (NSAIDs) such as aspirin (79). It can inhibit platelet activation (80) and may decrease the risk of IHD in subjects affected by IBD (11). Indeed, in a large Danish nationwide population-based study of Rungoe et al., the risk of IHD was lower among IBD patients using 5-ASA (IRR = 1.16; 95% CI: 1.06–1.26) than among non-users (IRR = 1.36; 95% CI: 1.22–1.51) (*p* = 0.02). Furthermore, long-term users of 5-ASA showed an even lower risk of IHD (IRR = 1.08; 95% CI: 0.98–1.19) (46). On the other hand, in the same study, the authors found that the subjects requiring oral corticosteroids had a higher risk of IHD (IRR = 1.37; 95% CI: 1.25–1.50) compared to the subjects who never used corticosteroids (IRR = 1.23; 95% CI: 1.12–1.36) (*p* < 0.01) (46). In fact, corticosteroids may exacerbate the risk of cardiovascular disease in subjects with IBD since they can aggravate some traditional risk factors such as hypertension, obesity, hyperlipidemia and insulin resistance (32). In another retrospective study, subjects affected by CD (but not UC) exposed to a prolonged assumption of corticosteroids were associated with increased rates of myocardial infarction compared to anti tumor necrosis factor (TNF)-alfa use (81). However, corticosteroids can be a proxy for disease severity, which itself is associated with incremented risk of IHD (46) and a clear causal association cannot entirely be determined (11).

Regarding TNF inhibitors, studies suggest a potential protective role of anti TNF-alfa drugs toward atherosclerosis and IHD (82). In an *in vitro* experiment, infliximab showed an atheroprotective effect by restoring the reverse cholesterol transport proteins which counteract foam cell formation by ridding cells of excess cholesterol (83). These findings appear to be confirmed also in clinical studies.

In a large population-based nationwide cohort of IBD patients, the exposure to anti-TNFs was associated with a decreased risk of acute arterial events (AAEs) compared to non-exposure (HR: 0.79, 95% CI: 0.66–0.95) (84). Furthermore, exposure to anti-TNFs was associated also with a decreased risk of recurrent acute arterial events (HR: 0.75, 95% CI: 0.63–0.90) in IBD patients with a previous history of AAEs (85). Thiopurines as well seem to possess an atherosclerotic protective feature since they also were associated with a decreased risk of recurrent AAEs (85). In addition, dos Santos et al. demonstrated that the use of azathioprine in association with 5-ASA, more than 5-ASA alone, can control the production of anti-inflammatory cytokines such as TGF-beta and IL-10, which participate to the modulation of the endothelial cells activation (86).

In regard to the more recently approved biologic agents for IBD (i.e., vedolizumab, ustekinumab), data concerning their role on AAEs are scarce (11). Data from clinical trials and post-marketing safety reports did not observe any safety signal of an augmented risk of AAEs in subjects with IBD treated with vedolizumab or ustekinumab (87–91) and no changes were observed in the serum lipid levels during the treatment with this classes of drugs (92).

Finally, Janus kinase inhibitors (JAK) (i.e., tofacitinib, filgotinib, upadacitinib) are part of one of the most recent class of drugs approved for the treatment of moderate-severe UC (93–95). In clinical studies, treatment with tofacitinib has been associated with generally reversible increases in serum lipids during the first few months of therapy, particularly in total cholesterol, high-density lipoprotein cholesterol and low-density lipoprotein cholesterol (96–102). Hypercholesterolemia is a risk factor for cardiovascular events and the potential occurrence of major adverse cardiovascular events (MACE) with has long been a concern (11). In the Oral Rheumatoid Arthritis Trial (ORAL) Surveillance study, a randomized safety endpoint trial which involved active rheumatoid arthritis (RA) patients who were 50 years of age or older with the presence of at least one cardiovascular risk factor and that compared combined tofacitinib doses (5 mg BID and 10 mg BID) and TNF inhibitor, the incidences of MACE (death from cardiovascular causes, nonfatal myocardial infarction, or nonfatal stroke) were higher in the tofacitinib groups than TNF inhibitor (HR: 1.33; 95% CI: 0.91–1.94) (103). As a result, the European Medicines Agency (EMA)'s human medicines committee (CHMP) has endorsed the measures recommended by the Pharmacovigilance Risk Assessment Committee (PRAC) which stated that tofacitinib (and all approved JAK inhibitors) should be used only if no suitable treatment alternatives are available in patients at increased risk of major cardiovascular problems (such as heart attack or stroke) (104). However, in the setting of UC, a long-term extension study of the OCTAVE open observed an incidence rate (IR) for MACE of 0.16 (95% CI: 0.04–0.42) among all patients (2,440.8 patient-years of exposure) (105). This result is in line with the IR of MACE observed with the use of TNF inhibitors in subjects with UC (incidence rate: 0.51, 95% CI: 0.31–0.79) (11). In addition, also real-life studies observed that tofacitinib did not confer a significantly elevated risk for MACE compared to TNF inhibitors (106, 107). Nevertheless, consistent with the ORAL surveillance trial, in the previously reported long-term extension study of the OCTAVE open the four adjudicated MACE occurred in patients aged ≥55 years, and three of the four subjects had a prior medical history that included risk factors for cardiovascular disease (105).

**Figure 2:**
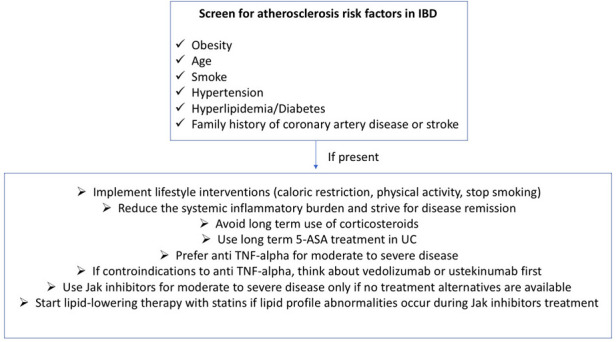
Recommendations for the management of atherosclerotic cardiovascular risk in IBD.

Less data are available on the cardiovascular safety profile of filgotinib or upadacitinib. In the registrative trials, events of MACEs were reported infrequently and no difference was observed with placebo (94, 95). Furthermore, in a meta-analysis assessing the safety of JAK inhibitors in subjects with either IBD or other immune-mediated inflammatory diseases (IMIDs), 30 studies evaluated MACEs on 32,765 patients exposed to JAK inhibitors (17 tofacitinib; 6 upadacitinib; 4 baricitinib; 3 filgotinib). The incidence rate was 0.67 per 100 patient-years. In addition, in the pooled analysis of 22 controlled studies, the RR of MACEs was 1.07 (0.56–2.03) (108). To our best knowledge, real-life safety data of filgotinib or upadacitinib in UC are missing. Table 3 summarizes the relationship between IBD therapies and atherosclerotic cardiovascular disease while Figure 2 provides recommendations for the management of the atherosclerotic risk in subjects with IBD.

**Table 3:** Relationship between IBD therapies and atherosclerotic cardiovascular disease.

IBD therapies	Effects on cardiovascular system
5-aminosalicylic acid (5-ASA)	↓ platelets activation
	↓ risk of ischemic heart disease
Corticosteroids	↑ risk of ischemic heart disease (insufficient data; corticosteroids can be a proxy for disease severity)
Thiopurines	↓ atherosclerosis and arterial events
TNF inhibitors	↓ atherosclerosis and arterial events
Vedolizumab or ustekinumab	Limited data. No increased risk of arterial adverse events in clinical trials and post-marketing safety reports
Anti JAK (tofacitinib, filgotinib, tofacitinib)	Not associated with an increased risk of major adverse cardiovascular events

Finally, in regards of medical prevention of atherosclerotic cardiovascular disease, even if the atherosclerotic risk is increased in IBD and although low-dose aspirin is not contraindicated in subjects with IBD and does not seem to increase disease flares (32, 109, 110), to date the indication for low-dose aspirin in either primary or secondary prevention in IBD does not change from those in general population (32). Concerning the drug category of statins, which are used for primary and secondary prevention of cardiovascular diseases, it has been suggested that this class of drugs may also exert complex immunomodulatory properties and might be beneficial agents for inflammatory conditions (111). However, even though statins can decrease inflammation in animal models of colitis (112), clinical studies evaluating their disease-modifying and preventive efficacy in IBD have shown conflicting results, being insufficient to support their use for preventing or treating IBD (111, 113, 114).

## 7. Conclusions

Increasing evidence suggests that IBD patients harbor an increased risk of arterial thrombotic events, especially the young subjects with active disease. This presumably may not be explained by traditional cardiovascular risk factors, but rather by chronic systemic inflammation. Therefore, the control of intestinal inflammation is an important outcome to reduce the risk of atherosclerosis and cardiovascular events. Generally, exposure to IBD's therapies does not seem to be associated with an increased risk of mayor adverse cardiovascular events. On the other hand, some classes of drugs, especially TNF inhibitors and azathioprine, may reduce the risk of arterial thrombotic events. JAK inhibitors are an important extension of the armamentarium against IBD and, unlike in other IMIDs, they do not seem to confer an additional risk of mayor adverse cardiovascular events. Further studies are needed to assess their cardiovascular safety profile in IBD.
